# Risk factors for digital dermatitis in free‐stall‐housed, Canadian dairy cattle

**DOI:** 10.1002/vro2.19

**Published:** 2021-08-02

**Authors:** Ellen de Jong, Klaas Frankena, Karin Orsel

**Affiliations:** ^1^ Department of Production Animal Health Faculty of Veterinary Medicine University of Calgary Calgary Canada; ^2^ Adaptation Physiology Group Wageningen Institute of Animal Sciences Wageningen University & Research Wageningen The Netherlands

**Keywords:** control strategies, dairy cattle, digital dermatitis, lameness, risk factors

## Abstract

**Background:**

A comprehensive analysis of the relation between digital dermatitis (DD) and cow and herd characteristics in Canadian dairies is currently lacking.

**Methods:**

A multilevel logistic regression analysis was performed using 12,260 cow records from 62 dairy farms to assess association between 27 cow and herd‐level variables, and presence of DD.

**Results:**

The odds for a cow to have at least 1 DD lesion were higher in first‐parity cows and those in later lactation (≥45 days in milk). Housing cows on a concrete base was associated with higher odds (OR 2.24) for DD when bedding was added once a week or less. Bedding the concrete base more frequently reduced odds for DD. Wood shavings or other bedding types were more positively associated with DD (OR 2.31 and 1.87, respectively) compared to sawdust. Also, the odds of DD were lower on farms with a scraping manure frequency of every 2 h compared to less frequent scraping (OR 0.54).

**Conclusion:**

Nine risk factors for DD were identified and quantified, with stall base, bedding type, and manure scraping frequency associated with lower odds of DD. DD prevalence could be reduced by implementing management practices for first‐parity cows, as they had higher odds of DD.

## INTRODUCTION

Digital dermatitis (DD) was first reported by Cheli et al.^1^ and is currently considered the main contributor to lameness cases of infectious origin in dairy cattle worldwide.^2^ DD lesions are considered to originate from bacterial infections, with treponemes being most commonly isolated.^3^ More than any other foot disorder, DD severely decreases animal welfare,^4^ and leads to economic losses related to reduced milk production and increased culling.^5^ Canadian free‐stall dairy farms have a herd prevalence estimated to range from 92.1% to 93.6% and a within‐herd DD prevalence ranging from 0 to 74.3%.^6^, ^7^

Numerous factors related to the individual cow and herd characteristics are suggested to affect DD prevalence. For instance, an association between the presence of DD and lactation stage in Holstein‐Friesian cows was reported, with fresh cows having lower odds of having DD compared to cows at peak or in late lactation,^8^ although the magnitude of this effect varied amongst parities.^7^ Additionally, presence of DD has been positively associated with first‐parity cows compared to multiparous cows; therefore, odds gradually decreased as parity increased.^7^, ^8^, ^9^ Concerning flooring, cows housed on slatted floors had higher odds of DD compared to those housed in straw yards (OR 11.1)^10^ or on non‐slatted concrete floors (OR 1.32).^11^ In addition, cows housed on grooved concrete had higher odds compared to textured concrete^12^ or non‐grooved concrete^11^ (OR 2.7 and 11.31 respectively). Reduced leg cleanliness has also been associated with a higher DD prevalence.^13^, ^14^ Frequent removal of manure from alleyways is therefore recommended, although both positive and negative effects on DD have been reported.^15^, ^16^ Whether or not associations among floor type, manure scraping frequency and presence of DD are causal associations has not been reported, but it is hypothesized that insufficient drainage of manure, along with muddy and moist conditions, reduced cleanliness and increased odds for DD.^17^, ^18^

Studies in the Netherlands,^8^, ^9^, ^10^ England and Wales^11^ and Chile^19^ have performed an integrated assessment of risk factors combining both cow factors (parity, milk production, lactation stage) and relevant management factors (stall base, floor type, bedding). However, results of those studies cannot be directly extrapolated to Canadian herds, due to major differences in production parameters, housing systems, herd sizes and climate. For example, the greatest adoption of automated milking systems is in European countries, where most automatic milking systems are manufactured as well, whereas adoption in Canada is significantly lower.^20^ In addition, outdoor access is not common for lactating cows, especially in Alberta; therefore, studies conducted on farms with seasonal pasture access^19^ are not applicable.

In Alberta, hoof trimming data were collected between 2009 and 2012 on 156 farms and included an assessment of cow factors.^7^ Between 2011 and 2012, 81 farms were visited and the relation between lameness and a large variety of herd management factors was assessed.^21^ Seventy‐six farms participated in both studies; therefore DD lesion data, as well as extensive herd and cow‐level data are available. As an overview, the current study aimed to use these existing data to simultaneously evaluate impacts of both cow and herd factors on the presence of DD in dairy cows permanently housed in free‐stall barns in Alberta. This comprehensive assessment of the most important risk factors for DD is key to further research into causal pathways and will contribute to optimizing management and control strategies to reduce occurrence of DD lesions.

## MATERIALS AND METHODS

### Study design

Two existing datasets were combined to address the study objectives. Dataset 1 contained hoof lesion data from computerized hoof trimming records collected by seven hoof trimmers and milk records (i.e. milk production, parity, lactation stage, herd size) provided by CanWest DHI (Guelph, ON, Canada) of 28,607 cows from 156 farms, collected from 2009 to 2012.^7^ Dataset 2 contained data on management practices and facility design factors from 81 farms, visited from 2011 to 2012,^21^ of which 5 farms were not covered in Dataset 1. The combined final dataset contained observations on 17,169 cows from 76 farms.

The individual study designs have been described in more detail in their respective publications.^7^, ^21^ In short, farms in this study were recruited via mail, telephone and fax. Eligibility criteria comprised a free‐stall barn, a herd size of >40 lactating Holstein‐Friesian cows, enrolment in the CanWest DHI (now Lactanet) recording system and participation in the Alberta Dairy Hoof Health Project (developed by Alberta Milk, Edmonton, AB, Canada). Data collection was approved by the Animal Care Committee (SHC10R‐07) and Research Ethics Board CFREB (file #6717).

### Data collection

For 3 years (2009 to 2012), hoof trimmers recorded the presence of active DD lesions (M2 lesions according to the M‐stage classification system) of cows presented to the trimmer on 76 farms, according to the Lesion Severity Scoring Guide,^22^ as part of either a whole‐herd (≥80% of lactating cows trimmed at once) or partial‐herd trimming (i.e. <80% of lactating cows trimmed; selections were done by the farmer). DD data were recorded in Hoof Supervisor lesion recording software (KS Dairy Consulting, Dresser, WI, United States) and DHI data of first available day after the trim date were obtained for each trimmed cow from CanWest DHI (Guelph, ON, Canada) with the consent of farmers. To ensure uniform recording of DD, two training sessions were held beforehand with the seven participating hoof trimmers. Although it was impossible to determine intra‐ and interobserver agreement among hoof trimmers, DD observations were not clustered within hoof trimmers, as the intraclass correlation was estimated to be very low (0.03).^7^

Farm characteristics were collected by trained observers during a farm visit between May 2011 and June 2012, during which a questionnaire was used as a guide for the study personnel to obtain details on practices related to footbath protocol, manure scraping system, and bedding change frequency. In addition, a checklist was used to assess feed alley floor type, feed alley cleanliness, feed alley slipperiness, and stall design (base, bedding, and dimensions), based on standard operating procedures developed and validated by Dairy Farmers of Canada.^23^

### Data management

All data management and statistical analyses were done using RStudio version 3.4.1.^24^ Since the trim dataset contained a combination of single and multiple cow records and because an opportunistic sampling strategy was followed, prevalence was calculated instead of incidence. Data management has been described.^7^ Briefly, records of cows that participated in multiple trim sessions were reduced to a single cow observation. For farms with a whole‐herd trimming strategy, the session with most unique cattle was chosen, whereas for farms with partial‐herd trims, the first trim record of each cow was chosen. Next, the trim record of each individual cow was linked to DHI data (parity, lactation stage and milk yield) of the most recent record after the trim session and merged with farm‐level characteristics of the second dataset. Duplicate observations were removed. A cow was considered positive for DD if at least 1 DD lesion was recorded. The within‐herd prevalence of DD was corrected for the proportion of the herd trimmed, as the proportion of the herd trimmed could deviate from the average herd size.

To assure sufficient power for statistical analyses, ordinal and nominal variables were critically reviewed, and categories were merged wherever necessary to yield sufficient observations per category.^25^ All continuous variables were checked for outliers (a value extraordinarily big or small in the absence of a valid explanation), which were removed from the dataset. Subsequently, continuous variables were checked for linearity in their log‐odds, as described by Hosmer et al.^26^ Variables with non‐linear log‐odds were categorized in groups, either data‐driven or based on literature. Missing values were classified as ‘not available’ and remained in the data set.

### Statistical analyses

The relationship between each independent variable and the presence of at least one DD lesion was examined in univariable logistic regression models at cow‐level, with farm as a random effect (Table S1), using the package ‘lme4’.^27^ Variables with *P* < 0.25 were retained and entered in various correlation matrices: Kendall rank for ordinal variables, Cramer's V for nominal variables, and Phi for dichotomous variables. The arbitrary threshold of *r = *0.60 was used to identify possible correlations. Of the highly correlated variables, the 1 with the highest univariable *P*‐value was selected when no biological preference was present to avoid co‐linearity in the model. Selected variables were introduced in a multivariable logistic regression model, with farm as a random effect.

In order to reach the most parsimonious model, a backward procedure was performed, where non‐significant (*P* > 0.05) variables were removed from the model 1 at a time, starting with the highest *P*‐value. If removal of a variable resulted in a relative change of >25% in any regression coefficient or an absolute change of >0.1 if the regression coefficient had a value between −0.40 and 0.40, it was considered a confounder and was retained in the model.^28^ Thereafter, all biologically relevant 2‐way interactions were tested; these were assessed 1 by 1 in separate models, instead of adding them all to the main model to prevent convergence problems. Interactions that resulted in a reduction in the Akaike information criterion (AIC) compared to the model without the interaction term,^25^ were then added to the main model.

Next, variables that were discarded during the selection process were tested in separate models to assess whether there was a significant relationship with the outcome variable in presence of the other variables, or if the variable was a confounder for any variable in the main model. Variables that were either significant or confounders remained in the final model. Afterwards, the backwards selection procedure was repeated, including testing additional interactions. From the final model, odds ratios (OR) were calculated alongside their 95% confidence interval (CI).

## RESULTS

In accordance with provincial averages, mean herd size was 183 lactating cows (74–517 cows) and mean 305‐day milk yield was 10,345 kg (8263–12,369 kg). Mean lactation length at time of trimming was 175 days in milk (83–231 days). Of the 76 farms, 29 trimmed the whole herd at each trim session, whereas the remaining 47 farms had a partial herd‐trimming strategy. Of the cows in the study population, 20.5% had at least one case of DD; 96.1% of the farms had at least one cow with DD; and within‐herd prevalence ranged from 0 to 74.3%.

Of the 17,189 cows recorded, 12,260 records from 62 farms were used in the final multilevel logistic regression model. Removal of 4929 records was largely attributed to having no DHI data available (*n* = 522), missing 305‐day milk yield estimates (*n* = 3143), and incomplete footbath data (7 farms, *n* = 733). Farm as a random effect accounted for 7% of the unexplained variation (likelihood ratio test compared to the model without a random effect: *χ* ^2^ = 238.78, 1 *df*, *P* < 0.001). Hoof trimmer was not included as a random effect as it did not explain any additional variation (likelihood ratio test compared to a model with only farm as a random effect: *χ* ^2^ = 0.00005, 1 *df*, *P* = 0.981); furthermore, it did not affect regression coefficients nor their confidence intervals. Three variables were not considered for the construction of the statistical model, 2 due to high correlations (305‐day milk yield was correlated with 24‐h milk yield and manure scraping system was correlated with manure scraping frequency) and 1 because there were limited observations in 1 group (bedding cleanliness). Nineteen 2‐way interactions were tested, of which stall base * bedding frequency remained in the final model. The distribution of the farms and cows among the variables of the final model are described in Table 1.

**TABLE 1:** Distribution of variables included in the multilevel logistic regression analysis. In total, 12,260 cows from 62 dairy farms in Alberta, Canada were included

		Frequency				
		Farm		Cow		
Variable	Category	n	%	n	%	Prevalence (% cows with DD)
Parity	1st parity			6,656	54	21.4
	2nd parity			2,721	22	22.3
	3rd parity			1,462	12	17.2
	4th parity			789	6	11.5
	≥5th parity			632	5	6.6
Lactation stage	Fresh (1‐45 days in milk)			2,863	23	15.8
	Peak (45‐100 days in milk)			2,197	18	19.6
	Mid (100‐200 days in milk)			3,229	26	21.5
	Late (≥200 days in milk)			3,971	32	21.2
24‐hour milk yield	<20 kg			993	8	17.1
	20–30 kg			3,977	32	18.6
	30–40 kg			4,938	40	21.1
	40–50 kg			1,876	15	21.1
	≥50 kg			473	4	15.2
Herd size	≥200 lactating cows	20	32	7,448	61	23.1
	100–200 lactating cows	38	61	4,482	37	13.6
	0‐100 lactating cows	4	6	330	3	27.0
Trim strategy	Whole herd trim	24	39	9,649	79	2.9
	Partial herd trim	38	61	2,611	21	82.1
Stocking density	<0.9 cows per stall	21	34	4,003	33	21.1
	0.9‐1.0 cows per stall	21	34	3,864	32	22.8
	≥1.0 cows per stall	20	32	4,393	36	15.7
Number of footbath products	1	19	31	3,738	30	23.6
	2	36	58	6,689	55	16.2
	≥3	7	11	1,833	15	24.7
Footbath width	<70 cm	25	40	4,587	37	17.2
	≥70 cm	37	60	7,673	63	21.2
Stall base * bedding frequency	Mattress * once a week or less	30	48	5,751	47	20.6
	Concrete * once a week or less	5	8	1,152	9	24.7
	Other^a^ * once a week or less	5	8	684	6	10.5
	Mattress * more than once a week	14	23	2,453	20	21.8
	Concrete * more than once a week	4	6	1,677	14	13.5
	Other^a^ * more than once a week	4	6	543	4	21.2
Bedding frequency * stall base	Once a week or less * mattress	30	48	5,751	47	20.6
	More than once a week * mattress	14	23	2,453	20	21.8
	Once a week or less * concrete	5	8	1,152	9	24.7
	More than once a week * concrete	4	6	1,677	14	13.5
	Once a week or less * other^a^	5	8	684	6	10.5
	More than once a week * other^a^	4	6	543	4	21.2
Bedding type	Sawdust	18	29	3,793	31	18.6
	Wood shavings	30	48	5,557	45	20.2
	Other^a^	14	23	2,910	24	20.3
Feed alley floor type	Slatted concrete	7	11	1,840	15	33.4
	Concrete	49	79	9,795	80	17.9
	Rubber	6	10	625	5	8.5
Feed alley slipperiness	No slipping	34	55	6,251	51	16.2
	Slipping	28	45	6,009	49	23.4
Feed alley cleanliness	Clean	42	68	7,475	61	18.8
	Dirty	20	32	4,785	39	21.1
Manure scraping frequency	Every hour	10	16	2,085	17	20.3
	At least every 2 hours	15	24	2,714	22	15.4
	At least 4 times a day	26	42	4,178	34	18.3
	Less than 4 times a day	11	18	3,283	27	24.7

^a^ Other stall bases included waterbed, deep‐bedded sand and composed manure.

The OR of the variables in the final model and their 95% CI are presented in Table 2. Six variables (i.e. 24‐h milk yield, number of footbath products used, footbath width, feed alley floor type, feed alley slipperiness, and feed alley cleanliness) were non‐significant, but had a confounding effect on the estimates for, among others, herd size and manure scraping frequency and thus were retained in the model.

**TABLE 2:** Odds ratios of variables included in the multilevel logistic regression analysis with digital dermatitis as the outcome variable, and farm as a random effect (12,260 cows on 62 farms in Alberta, Canada)

				*P*‐value	
Variable	Category	Odds ratio	95% CI	Category	Variable
Parity	1st parity	Referent			<0.001
	2nd parity	0.97	0.86‐1.10	0.662	
	3rd parity	0.73	0.62‐0.87	<0.001	
	4th parity	0.45	0.35‐0.57	<0.001	
	≥5th parity	0.23	0.16‐0.32	<0.001	
Lactation stage	Fresh (1‐45 days in milk)	Referent			<0.001
	Peak (45‐100 days in milk)	1.26	1.07‐1.48	0.005	
	Mid (100‐200 days in milk)	1.48	1.28‐1.72	<0.001	
	Late (≥200 days in milk)	1.47	1.27‐1.70	<0.001	
24‐hr milk yield^a^	<20 litre	Referent			0.140
	20–30 litre	1.09	0.90‐1.33	0.377	
	30–40 litre	1.21	0.99‐1.47	0.066	
	40–50 litre	1.28	1.01‐1.61	0.042	
	≥50 litre	1.06	0.74‐1.50	0.753	
Herd size	≥200 lactating cows	Referent			0.001
	100–200 lactating cows	0.50	0.34‐0.73	<0.001	
	0‐100 lactating cows	0.85	0.38‐1.87	0.682	
Trim strategy	Whole herd trim	Referent			<0.001
	Partial herd trim	2.26	1.52‐3.36	<0.001	
Stocking density	<0.9 cows per stall	Referent			<0.001
	0.9‐1.0 cows per stall	2.18	1.42‐3.34	<0.001	
	≥1.0 cows per stall	0.72	0.48‐1.08	0.114	
Number of footbath products used^b^	1	Referent			0.210
	2	0.66	0.42‐1.05	0.080	
	≥3	0.84	0.47‐1.50	0.542	
Footbath width^b^	<70 cm	Referent			0.073
	≥70 cm	1.40	0.97‐2.03	0.073	
Stall base * bedding frequency	Mattress * once a week or less	Referent			0.001
	Concrete * once a week or less	2.23	1.22‐4.09	0.009	
	Other * once a week or less	0.37	0.17‐0.77	0.008	
	Mattress * more than once a week	Referent			0.099
	Concrete * more than once a week	0.45	0.20‐0.99	0.048	
	Other * more than once a week	0.56	0.27‐1.19	0.131	
Bedding frequency * stall base	Once a week or less * mattress	Referent			0.335
	More than once a week * mattress	1.25	0.79‐1.96	0.335	
	Once a week or less * concrete	Referent			0.002
	More than once a week * concrete	0.25	0.11‐0.60	0.002	
	Once a week or less * other	Referent			0.165
	More than once a week * other	1.93	0.77‐4.79	0.165	
Bedding type	Sawdust	Referent			<0.001
	Wood shavings	2.31	1.48‐3.59	<0.001	
	Other	1.87	1.17‐2.99	0.009	
Feed alley floor type^c^	Slatted concrete	Referent			0.011
	Concrete	0.42	0.22‐0.80	0.008	
	Rubber	0.24	0.09‐0.63	0.004	
Feed alley slipperiness^d^	No slipping	Referent			0.152
	Slipping	0.75	0.51‐1.11	0.152	
Feed alley cleanliness^e^	Clean	Referent			0.060
	Dirty	1.45	0.99‐2.14	0.060	
Manure scraping frequency	Every hour	Referent			0.003
	At least every 2 hours	0.84	0.42‐1.67	0.524	
	At least 4 times a day	1.84	1.07‐3.17	0.018	
	Less than 4 times a day	0.57	0.35‐0.95	0.115	

^a^ Confounder for lactation stage, herd size and feed alley floor type.

^b^ Confounder for herd size and manure scraping frequency.

^c^ Confounder for feed alley slipperiness, number of footbath products used, manure scraping frequency and stall base.

^d^ Confounder for herd size, footbath width, number of footbath products used, bedding type and manure scraping frequency.

^e^ Confounder for herd size, stocking density, number of footbath products used and manure scraping frequency.

^f^Other stall bases included waterbed, deep‐bedded sand and composed manure.

Parity was significantly associated with the presence of DD. Cows in their third, fourth and fifth or higher parity had lower odds compared to cows in their 1^st^ parity (OR 0.73, 0.45 and 0.23 respectively). Cows in peak, mid or late lactation had higher odds for DD (OR 1.26, 1.48 and 1.47 respectively) compared to early lactation cows. The partial herd (< 80% of the lactation cows) trimming strategy was associated with higher odds for DD compared to farms that trimmed the whole herd (OR 2.26). Both stall base and bedding frequency were associated with DD and there was an interaction between the 2. When new bedding was added once a week or less, cows housed in a concrete stall base had higher odds for DD compared to a mattress as stall base (OR 2.23). However, an opposite effect existed when bedding was added more than once a week. In that regard, cows housed in stalls with a concrete base and ‘other’ base had lower odds (OR 0.45). Regardless of stall base and bedding frequency, the use of either wood shavings or ‘other’ (waterbed, composed manure and sand) bedding types resulted in higher odds for DD compared to sawdust as bedding material (OR 2.31 and 1.87 respectively). Finally, cows on farms where manure in the alleys was scraped at least 4 times a day had an OR of 1.84 compared to cows in farms where manure was scraped every hour.

## DISCUSSION

This comprehensive analysis of simultaneously assessing the relation between DD and cow and herd characteristics is the first of its kind in Canada. Parity, lactation stage and several other factors related to the direct environment of the cow (stall base, bedding type, bedding frequency, and manure scraping frequency) influenced the odds for a cow to have at least 1 DD lesion.

The identification of DD lesions was performed by seven hoof trimmers and active DD lesions, known as M2,^29^ were recorded by trimmers as part of their normal routine. Though differences between trimmers in the recording of DD lesions may have existed, the intraclass correlation coefficient of observations among the hoof trimmers was very small (0.03) indicating that scores were not affected by differences between trimmers. Cows that were presented to the hoof trimmer were, in 61% of the herds, selected by the producer as they used a partial herd trim strategy. Consequently, animals deemed at higher risk such as (suspected) lame animals, had a higher probability of being selected for trimming compared to non‐lame cows. Therefore, trim strategy and presence of a DD lesion were associated (OR 2.26) as previously reported by Solano et al.^7^ As a result, the identified risk factors might therefore not be entirely representative for cows with DD that do not have increased risk.

A prevalence approach was chosen over an incidence approach, due to the opportunistic nature of the data collection, where a baseline hoof health assessment of each individual animal was absent. The animal data of the current study is a subset of Solano et al.,^7^ who reported a similar DD herd‐prevalence (96.1%), cow‐prevalence (20.5%) and within‐herd prevalence (ranging from 0 to 74.3%). However, due to the less frequent trimming in herds with a whole herd trim strategy, some active DD lesions might have become chronic at time of trimming. Hoof trimmers only recorded active lesions, also known as M2 lesions.^29^ Chronic stages of DD lesions were not captured in this study, which could have resulted in an underestimation of the actual prevalence of DD lesions in Alberta.

The lower odds for DD as parity increased were consistent with results from studies in The Netherlands,^8^, ^9^ although results are not directly comparable as genetic selection might have improved hoof health outcomes, and herd sizes in the early 2000s averaged 50–60 cows,^30^ which is smaller than average herd size in the current study (183 cows). The lower odds could be attributed to increased risk of lame cows being culled,^31^ although no culling information was available to verify this assumption. The relation between lactation stage and DD, where cows in later lactational stages had higher odds for DD, was similar as for dairy cattle in Chile.^19^ Although 2 other studies^7^, ^8^ mentioned an association between lactation stage and DD, these results could not be easily compared to outcomes of the current study, due to an interaction‐effect with parity. The exact way via which lactation stage is related to DD can only be hypothesized. DD lesions might have been present early in lactation, but not yet detected, as it can take on average 133 days until formation of active lesions.^32^

Consistent with other reports,^7^, ^12^, ^15^ a large herd size (≥200 lactating cows) was associated with increased odds for DD. Purchasing cows is a common practice in North America; larger herds are more likely to purchase cows, which could increase their odds of introducing diseases into the herd compared to smaller herds.^33^ The lack of a statistical difference between herds with 0–100 lactating cows and herds with ≥200 cows may be due to the small number of herds with <100 lactating cows (6 herds).

Although numerous combinations between stall base and bedding frequency were identified in this study, their relevance with respect to DD should be examined closely to rule out any spurious combinations. Associations between bedding type, stall base and lameness have been mentioned by others,^16^, ^21^, ^34^ but not specifically in relation to DD. Cook et al.^35^ suggested that stall surfaces that have fewer cushioning properties result in increasing standing time and therefore increased odds for lameness; however, such extrapolations should be interpreted with caution, as the presence of DD does not always result in clinical lameness.^36^ In this study, the increased odds for DD associated with the use of mattresses only applied to situations where the bedding was added once a week or less. When stalls were bedded more often, a concrete base appeared to be beneficial. This suggests a more frequent change of bedding material is recommended for a concrete stall base, compared to a mattress base.

Although both the interaction between bedding type and bedding quantity has been examined, and the interaction between stall base and bedding quantity, neither of these interactions was significant. The exact way in which bedding frequency has a role in combination with stall base thus remains unknown. With respect to bedding type, sawdust as bedding material was associated with lower odds for DD. Sawdust can absorb moisture and thus contributes to a dry and clean environment. Therefore, sawdust is recommended over wood shavings, regardless of the bedding frequency and specific stall base.

A high manure scraping frequency was associated with lower odds for DD and thus a scraping frequency of at least every 2 h is recommended. Less frequent manure removal can result in build‐up of slurry in the barn, creating unhygienic conditions that likely favour DD. Similarly, automatic scrapers on low frequency are also known to build up slurry in front which contributes to those unhygienic conditions,^37^, ^38^ although it was not possible to compare manual versus automatic manure scraping systems due to the correlation with scraping frequency.

Footbaths are commonly used to control DD within a herd as part of internal hygiene strategies as there is sufficient research indicating a positive effect of footbaths with copper sulphate and other products on the level of DD.^39^, ^40^, ^41^ The effectiveness of a standardized footbath protocol with copper sulphate in decreasing DD prevalence was studied in Alberta specifically and was found to be successful in herds with high DD prevalence.^39^ In the current study, the association between footbath variables and the presence of DD was not significant, although these variables remained in the final model as they acted as confounders for herd size and manure scraping frequency.

The influence of biosecurity measures on the occurrence of DD lesions was not analyzed in this study as no data on biosecurity measures were collected. Considering the bacterial origin of DD lesions, biosecurity is an important consideration of DD control strategies. Treponemes are most commonly isolated from DD lesions^3^ and can persist on hoof trimming equipment if not properly cleaned.^42^, ^43^ Increased prevalence has been associated with the use of outside staff for hoof trimmers,^44^, ^45^ implying that these bacteria can be transmitted between herds. A higher DD prevalence has also been reported on farms that lacked boots for visitors and farm staff working at other dairy farms,^15^ and farms that purchase cows and heifers,^9^, ^43^, ^46^ emphasizing the importance of good hygiene practices.

In conclusion, this study identified and quantified nine risk factors related to the individual cow and herd characteristics, for example environment and management decisions. Proper stall base, bedding type and manure scraping frequency have the potential to significantly reduce the odds for DD. In addition, first‐parity cows should be monitored closely as they were classified as having higher odds for DD. Knowledge of these risk factors and associated recommendations will inform research into causal pathways and aid the development of reasonable and easy to implement management and control strategies aimed at preventing DD lesions.

## AUTHOR CONTRIBUTIONS

KO contributed to the study design. EdJ performed the analysis. KF and KO verified the analytical methods. EdJ drafted the manuscript. All authors read, edited and approved the final manuscript.

## CONFLICT OF INTEREST

The authors have declared no conflict of interest.

## ETHICS APPROVAL

This study was approved by the University of Calgary Animal Care Committee (SHC10R‐07) and Research Ethics Board CFREB (file #6717).

## Supporting information

SUPPORTING INFORMATION

SUPPORTING INFORMATION

## Data Availability

The data that support the findings of this study are available from the corresponding author upon reasonable request.
