# Supplementary material for: Risk factors for digital dermatitis in free‐stall‐housed, Canadian dairy cattle
Source: Vet Rec Open. 2021 Aug 2;8(1):e19. doi: 10.1002/vro2.19 (PMC8330421; doi:10.1002/vro2.19)
Supplement: Supplementary file 1 — SUPPORTING INFORMATION [file VRO2-8-e19-s002.pdf]

SUPPLEMENTARY MATERIAL

Table S1: List of variables collected for the objective of this study, their collection method, definition, and their relation to the presence of digital dermatitis

Variable	Collection method[1]	Definition	*P*-value[2]
Parity	DHI	Parity (1st, 2nd, 3rd, 4th, ≥5th) on day of trimming	< 0.001
Lactation stage	DHI	Days in milk on day of trimming: fresh (1 – 45 days in milk), peak (45 – 100 days), mid (100 – 200 days) or late (≥ 200 days)	< 0.001
305-day milk yield	DHI	kg	
24-hour milk yield	DHI	Milk production on day of trimming: < 20kg, 20-30 kg, 30-40 kg, 40-50 kg, ≥50 kg	0.045
Herd size	HT	< 100, 100 – 200 or ≥ 200 cows in milk	0.001
Trim strategy	MQ	Whole herd or partial herd trim (< 80 % of lactating cows)	< 0.001
Stocking density	DHI	<0.9, 0.9-1.0 or ≥1/0 cows per stall	0.530
Number of products used for footbath	MQ	1, 2 or ≥ 3 number of different products used for footbath(s)	0.520
$CuSO_4$ present	MQ	Use of $CuSO_4$ as (one of the) footbath products? Yes or no	0.624
Formaldehyde present	MQ	Use of formaldehyde as (one of the) footbath products? Yes or no	0.202
Number of footbath sessions	MQ	How often a footbath is used. Every week or once per two/three weeks	0.843
Footbath length	BC	Length of the footbath in cm	0.590
Footbath depth	BC	< 15 cm or ≥ 15 cm	0.295
Footbath width	BC	< 70 cm or ≥ 70cm	0.173
Stall base	BC	Defined as surface under bedding. Concrete, geo/rubber mattress or other (waterbed, deep-bedded sand, composted manure)	0.410
Stall cleaning frequency	MQ	Once per day or less than once per day	0.579
Bedding frequency	MQ	Once or less than once per week, more than once per week	0.712
Bedding type	BC	Wood shavings, sawdust or other (straw, composted manure, sand)	0.700
Bedding quantity	BC	Measured at two spots near the back of the stall. Deep (> 2 cm) or little (≤2 cm)	0.970
Bedding dryness	BC	Estimated by kneeling onto paper towels placed in stall. Dry or wet	0.785
Feed alley floor type	BC	Slatted concrete, grooved/textured concrete or grooved/textured rubber	0.160
Feed alley slipperiness	BC	Measured in 20 cows as they walk towards milking parlor. Slipping (≥ 3% of the cows slip of ≥ 1% of the cows fall) or no slipping	0.034
Feed alley cleanliness	BC	Measured in front of the feed bunk. Dirty (> 3cm manure thickness), or clean (≤ 1 cm manure thickness)	0.248
Feed alley width	BC	Width of feed alley, < 4.0 m or ≥ 4.0 m	0.566
Manure scraping system	BC	Automatic or manual	
Manure scraping frequency	MQ	Every hour (=24 times), at least every 2 hours (>=12 times), at least 4 times a day (>=4 times), less than 4 times a day (<4 times)	0.810

[1] DHI = obtained via CanWest, HT = collected by hoof trimmer, MQ = response of farmer in management questionnaire, BC = observed by authors according to in-barn checklist

[2] Multilevel logistic regression analysis with farm as random effect, DD as outcome variable, and the respective variable as an explanatory variable
